# The role of age, gender, mood states and exercise frequency on exercise dependence

**DOI:** 10.1556/JBA.2.2013.014

**Published:** 2013-10-15

**Authors:** Sebastiano Costa, Heather A. Hausenblas, Patrizia Oliva, Francesca Cuzzocrea, Rosalba Larcan

**Affiliations:** ^1^Department of Human and Social Sciences, University of Messina, Messina, Italy; ^2^Department of Exercise and Sport Sciences, Jacksonville University, Jacksonville, FL, USA

**Keywords:** exercise dependence, age, gender, mood states, exercise

## Abstract

**Objectives:**

The purpose of our study was to explore the prevalence, and the role of mood, exercise frequency, age, and gender differences of exercise dependence. *Methods:* Regular exercisers (*N* = 409) completed a socio-demographic questionnaire, the Exercise Dependence Scale, and the Profile of Mood States. For data analyses, the participants were stratified for sex and age (age ranges = young adults: 18–24 years, adults: 25–44 years, and middle-aged adults: 45–64 years). *Results:* We found that: (a) 4.4% of the participants were classified as at-risk for exercise dependence; (b) the men and the two younger groups (i.e., young adults and adults) had higher exercise dependence scores; and (c) age, gender, exercise frequency, and mood state were related to exercise dependence. *Conclusions:* Our results support previous research on the prevalence of exercise dependence and reveal that adulthood may be the critical age for developing exercise dependence. These findings have practical implication for identifying individuals at-risk for exercise dependence symptoms, and may aid in targeting and guiding the implementation of prevention program for adults.

## INTRODUCTION

Current exercise guidelines emphasize the need to practice regular physical activity for its health-related benefits. These guidelines also recommend that an increased amount of exercise is associated with additional health benefits (USDHHS, 2008). No current guidelines exist, however, for when an increased amount of exercise becomes “too much” and thus has negative health outcomes. For example, not all the effects of sustained engagement with exercise are positive, and some individuals exercise excessively and may become addicted. Excessive physical activity manifests in physiological and psychological symptoms and it is generally defined as exercise dependence (Hausenblas & Symons Downs, 2002a).

To further our understanding of exercise dependence Hausenblas and Symons Downs (2002b) redefined and applied the following seven *DSM-IV-R* diagnostic criteria for substance dependence (American Psychiatric Association, 2000) to exercise: *tolerance, withdrawal, intention effects, lack of control, time, continuance,* and *conflict*. In this perspective, exercise dependence is described as a craving for exercise that results in uncontrollable excessive physical activity that manifests in physiological symptoms and/or psychological symptoms (Hausenblas & Symons Downs, 2002a, 2002c).

Although researchers have examined various aspects of exercise dependence, little is known about its epidemiology. Due to the serious physical, psychological, and social outcomes associated with exercise dependence symptoms it is important to examine the variables that contribute to its development, onset, and progression, such as age and gender (Adams, 2009; Adams, Miller & Kraus, 2003; Costa & Oliva, 2012). Identifying factors associated with exercise dependence is also important since it will help guide future research and the implementation of a prevention program across the lifespan.

Preliminary research has found varying prevalence rates for exercise dependence ranging from less than 10% to about 35% of regular exercisers being classified as exercise dependent (Allegre, Souville, Therme & Griffiths, 2006; Berczik et al., 2012; Costa, Cuzzocrea, Hausenblas, Larcan & Oliva, 2012; Garman, Hayduk, Crider & Hodel, 2004; Hausenblas & Symons Downs, 2002a; Lejoyeux, Avril, Richoux, Embouazza & Nivoli, 2008; Lindwall & Palmeira, 2011; Terry, Szabo & Griffiths, 2004; Villella et al., 2011; Zmijewski & Howard, 2003). The inconsistent prevalence rates may, in part, be related to the heterogeneity of measurement tools used to assess exercise dependence (Allegre et al., 2006). When researchers use the two most popular and psychometrically validated instruments, the *Exercise Dependence Scale* and the *Exercise Addiction Inventory* (which yield comparable results; Sussman, Lisha & Griffiths, 2011), the prevalence of exercise addiction ranges between 3% to 5% of the population (Berczik et al., 2012; Mónok et al., 2012). Most of the studies on exercise dependence, however, were completed primarily with college students, and for this reason limited information exists of the prevalence of exercise dependence in different age groups (Mónok et al., 2012; Sussman et al., 2011).

Similarly, inconsistent gender differences for exercise dependence symptoms (Hausenblas & Symons Downs, 2002a; Szabo, 2000) may also be a measurement artifact (Weik & Hale, 2009). Multidimensional measures of exercise dependence (such as the *Exercise Dependence Scale*; Hausenblas & Symons Downs, 2002a) have shown that men are more exercise dependent than women (Edmunds, Ntoumanis & Duda, 2006; Hausenblas & Fallon, 2002; Hausenblas & Symons Downs, 2002c), whereas other measures, such as the *Exercise Dependence Questionnaire* (Ogden, Veale & Summers, 1997), have found that women are more dependent than men; with women scoring higher than men on weight control related subscales (Kjelsås & Augestad, 2003; Zmijewski & Howard, 2003). An explanation for these conflicting results was provided by Adams (2009), who stated that the gender differences on the Exercise Dependence Questionnaire were most likely artifacts of the instrument design that includes a disordered eating behavior subscale.

Regarding the relationship between age and exercise dependence, most studies have examined college students. Depending on the sample size and the degree of sophistication of the research design, this can produce valuable information and insights regarding exercise dependence in general. However, the focus on college students and the absence of cross-sectional analyses from different age groups of exercisers makes it difficult to explore risk factors for exercise dependence prevalence by age. Szabo (2000) stated that the prevalence for exercise dependence should decline with age as older exercisers develop a more balanced lifestyle. Furthermore, because of the negative linear relationship between exercise and age, it is likely that a negative relationship also exists between exercise dependence and age with older adults being less at-risk for exercise dependence than their younger counterparts (Sussman et al., 2011). In support of this, Edmunds et al. (2006) found that younger participants (aged 34 years and younger) had higher exercise dependence scores than those over 35 years of age. In contrast, a more recent study on middle class weightlifters failed to find addictive behavior differences between young adults (18–24 years) and adults (25–55 years; Hale, Roth, DeLong & Briggs, 2010). These incongruent results show the necessity of further study on age differences for exercise dependence. In fact, previous studies have analyzed age differences in exercise addiction attributing participants in *ad hoc* age groups to balance the sample size, and for this reason the incongruence of results could be due to the age range.

Exercise dependence symptoms are positively correlated to the mood deterioration and the length of involvement in physical activity (Adams, 2009; Berczik et al., 2012). Mood states can play a role in either the development or maintenance of exercise dependence. Addicted exercisers are more depressed, anxious, tense, confused, and angry after missing a workout (Adams, 2009; Berczik et al., 2012; Hamer & Karageorghis, 2007). Negative mood states and intensive exercise can be factors that can increase exercise dependence (Berczik et al., 2012; Hamer & Karageorghis, 2007). The research, however, linking mood states, exercise behavior, and exercise dependence, has targeted adolescents, college-age students, or adult sub-groups without examining age in the link between these factors. Consequently, little is known about the role of mood states and exercise dependence across the adult lifespan.

Determining the association of exercise dependence symptoms with mood states and exercise frequency, controlling for age and gender groups across the adult lifespan is needed to understand the mechanism underlying the changes in exercise dependence symptoms during the adult lifespan. In fact, physical activity levels decrease gradually with age (Brunet & Sabiston, 2011; Haskell et al., 2007; Sallis, 2000), and older adults may be able to regulate their emotions more than younger adults (Birditt, Fingerman & Almeida, 2005; Magai, Consedine, Krivoshekova, Kudadjie-Gyamfi & McPherson, 2006). For this reason exercise frequencies and negative mood states are relevant factors in the development and maintenance of exercise dependence (Berczik et al., 2012; Hamer & Karageorghis, 2007; Hausenblas & Symons Downs, 2002a; Rocheleau, Webster, Bryan & Frazier, 2004) that could be influenced by age.

The purpose of our study was to examine the prevalence of exercise dependence and the role of gender, age, exercise frequency and mood on exercise dependence symptoms. We hypothesized that exercise dependence levels would vary according to age and gender groups, such that young adults and adults would report higher levels than middle-aged adults (Edmunds et al., 2006; Sussman et al., 2011; Szabo, 2000). Concerning gender, we hypothesized that men would report more exercise dependence symptoms than women (Berczik et al., 2012; Weik & Hale, 2009). Finally, in line with the role of mood states and exercise frequency in the development and maintenance of exercise dependence (Adams, 2009; Berczik et al., 2012), we hypothesized that younger age, male gender, high exercise frequency and negative mood states would be related to exercise dependence symptoms.

## METHODS

### Participants

Participants were 409 (*n* = 209 men and *n* = 200 women) regular exercisers who were categorized into the following 3 age categories: 18–24 years (young adults: 51 men and 50 women), 25–44 years (adults: 84 men and 81 women), and 45–64 years (middle-aged adults: 74 men and 69 women; Spirduso, Francis & MacRae, 2005). All the participants were Italian with an average body mass index (BMI) in the normal range (*M* = 23.95, *SD* = 3.29; 95% confidence interval, 23.63–24.27). Most of the participants were either single (40.8%) or married (40.8%), followed by divorced or separated (4.2%), living together with a partner (3.4%), and widowed (1%), (9.7% of participants did not answer this question). An *a priori* power analysis, conducted using G*Power (version 2; Faul & Erdfelder, 1992), ensured that the sample sizes were sufficient to yield adequate statistical power for the procedures conducted in our study. More specifically, to detect a significant finding (at the .05 level) at a desired power level of .95, a minimum of 125 participants were required with 35 participants in each of the subgroups.

### Procedure

Participants were recruited from 16 gyms from the city of Messina in Italy. All the participants had a history of regular exercise for at least 3 months. After describing the nature of the study, interested individuals voluntarily completed an institutionally approved informed consent. In a separate room of the gym the participants completed a 30-minute assessment that consisted of a series of self-reported questionnaires assessing socio-demographic, exercise frequency, exercise dependence, mood states, and eating disorder factors. A research assistant was present to monitor the participants’ progress and provide assistance. Of the 456 persons who signed the informed consent 32 failed to complete the entire questionnaire. To eliminate the confounding effects of secondary dependence, the 15 participants (4 men and 11 women) – who scored in the at-risk range on the Drive for Thinness subscale (14 or greater; Garner, 1991) of the Eating Disorder Inventory-2 – were excluded from the analysis. The following three age categories were formed: 18–24 years (young adults), 25–44 years (adults), and 45–64 years (middle-aged adults). This age-stratification was based on Spirduso et al.’s (2005) suggestion that physical activity plays a different role in each of these age groups. This stratified sampling has been widely used in several studies and national database (e.g., Adams & Schoenborn, 2006; Brunet & Sabiston, 2011; Ham & Ainsworth, 2010; Román-Viñas et al., 2007; Wilson, Elliott, Eyles & Keller-Olaman, 2007).

### Measures

*Socio-demographic questionnaire.* The socio-demographic questionnaire assessed the participants' age, gender, weight, and height. Self-reported height and weight was used to compute BMI.

*Exercise measure.* Data regarding exercise frequency and duration was developed according to Caspersen, Powell and Christenson (1985) definition of “exercise”. The question about frequency was, “How often do you exercise in a week?” The question about duration was, “How long do you usually exercise on each occasion?” (Adkins & Keel, 2005; Eriksson, Baigi, Marklund & Lindgren, 2008; Lindwall & Hassmén, 2004; Oh, Yoon & Shin, 2005). To produce a score for overall extent of exercise frequency, the number of days was multiplied by the number of hours.

*Drive for thinness.* The Drive for Thinness subscale is a 7-item subscale of the Eating Disorder Inventory-2 (Garner, 1991) that measures disordered eating attitudes about body image, weight, and shape. Participants indicate how often they agree with the statements concerning preoccupation with weight (e.g. “I am preoccupied with the desire to be thinner”) on a 6-point Likert scale ranging from 1 *(never)* to 6 *(always)*, with a higher score indicating a greater pursuit for thinness. The internal consistency in our study was good (alpha = .83). Individuals with scores greater than or equal to 14 on this subscale were considered to be at-risk for an eating disorder.

*Exercise dependence.* The Italian version of the Exercise Dependence Scale-R (EDS-R; Costa et al., 2012; Symons Downs, Hausenblas & Nigg, 2004) was used to measure exercise dependence symptoms. The EDS-R consists of 21 items scored on a 6-point Likert scale, ranging from 1 *(never)* to 6 *(always).* Higher scores indicate more exercise dependence symptoms. The instrument has seven subscales (three items for each) based on the *DSM-IV-TR* criteria for substance dependence and a total score. For our study coefficient alpha’s for the subscales were good (alpha range = .70–.94). We used the criteria established in the *DSM-IV-TR,* to classify individuals in the following exercise dependence groups: at-risk for exercise dependence (i.e., scores of 5–6 on average on the Likert scale in at least three of the seven criteria), nondependent symptomatic (i.e., scores of 3–4 on average in at least three criteria, or scores of 5–6 on average combined with scores of 3–4 on average in three criteria, but failing to meet the criteria of at-risk conditions), and nondependent asymptomatic (i.e., scores of 1–2 on average in at least three criteria).

*Mood states.* The Italian version of the Profile of mood states (POMS; Farnè, Sebellico, Gnugnoli & Corallo, 1991) was administrated to monitor mood states. This scale comprises 65 items scored on a five-point scale ranging from 0 to 4 and measures six subscales: tension–anxiety, depression–dejection, anger–hostility, vigor–activity, fatigue–inertia, confusion–bewilderment. The internal consistency scores in our study (see Table 3) were acceptable to good (alpha range = .78–.87).

### Ethics

The study procedures were carried out in accordance with the Declaration of Helsinki. The Institutional Review Board of the University of Messina approved the study. All subjects were informed about the study and all provided informed consent

## RESULTS

### Preliminary analyses

Information about the prevalence of exercise dependence (i.e., at-risk, symptomatic, and asymptomatic), age, BMI, and exercise frequency are presented in Table 1. Based on the EDS-R criteria, 18 (4.4%) participants were classified as at-risk, 230 (46.9%) as nondependent-symptomatic, and 161 (39.4%) as nondependent-asymptomatic. There were no significant gender differences in the number of participants classified at-risk for exercise dependence, χ^*2*^ (1) = 2.00, *p* = .15. For age, significantly more male adults (25–44 years) than young male adults (18–24 years) reported to be at-risk for exercise dependence, χ^*2*^ (1) = 5.44, *p* = .02. There were no age differences in the female groups for the prevalence of exercise dependence symptoms, χ^*2*^ (2) = 1.00, *p* = .61. Mean, standard deviation, alphas, skewness, and kurtosis values are listed in Table 2 for exercise dependence variables and Table 3 for the mood states variables.

**Table 1.** Descriptive statistics by age and gender

		18–24		25–44		45–64		
		Men	Women	Men	Women	Men	Women
		*n* =51	*n* =50	*n* =84	*n* =81	*n* =74	*n* =69
At-risk	*N*	1	2	8	3	3	1
	(%)	0.24%	0.49%	1.96%	0.73%	0.73%	0.24%
Symptomatic	*N*	40	28	48	43	41	30
	(%)	9.78%	6.85%	11.74%	10.51%	10.02%	7.33%
Asymptomatic	*N*	10	20	28	35	30	38
	(%)	2.44%	4.89%	6.85%	8.56%	7.33%	9.29%
Age	*M*	21.80	21.32	33.11	34.48	52.12	51.75
	*SD*	1.61	1.83	5.67	5.66	4.74	4.25
Body mass index	*M*	23.40	21.07	25.15	22.25	25.59	24.49
	*SD*	2.35	2.69	2.68	4.299	3.94	3.22
Exercise frequency	*M*	6.24	5.31	6.77	5.14	5.20	4.43
	*SD*	1.96	2.52	4.64	3.14	4.05	2.98

**Table 2.** Mean and standard deviation scores by age and gender for exercise dependence

	α	Skew	Kurt		18–24		25–44		45–64	
					Men	Women	Men	Women	Men	Women
Withdrawal	.79	.95	.39	*M*	6.43	7.46	6.27	7.14	6.32	6.16
				*SD*	3.25	3.37	3.40	3.57	3.82	3.19
Continuance	.74	1.18	.73	*M*	6.14	5.54	5.96	4.81	5.47	6.03
				*SD*	3.38	2.96	3.41	2.51	2.81	3.20
Tolerance	.81	.38	–.81	*M*	11.47	9.00	10.86	8.65	9.14	7.28
				*SD*	3.90	4.01	4.43	4.37	4.70	3.80
Lack of control	.85	1.04	.43	*M*	7.45	6.90	7.07	5.95	7.09	5.83
				*SD*	3.87	4.14	3.92	3.26	4.42	3.48
Reduction in other activities	.70	1.52	2.64	*M*	5.65	5.28	5.70	4.75	4.77	4.36
				*SD*	2.20	2.37	3.18	2.37	2.26	1.99
Time	.79	.71	–.24	*M*	9.41	8.10	9.81	7.98	7.80	6.51
				*SD*	2.97	4.01	4.23	4.29	4.06	3.05
Intention	.83	.84	–.02	*M*	7.88	7.18	8.20	7.23	7.32	5.64
				*SD*	3.46	3.89	4.15	3.97	4.14	3.38
Total exercise dependence	.94	.58	–.01	*M*	54.39	49.40	53.86	46.52	48.61	41.80
				*SD*	13.33	15.80	18.09	15.04	18.06	14.64

**Table 3.** Mean and standard deviation scores by age and gender for mood states

	α	Skew	Kurt		18–24		25–44		45–64	
					Men	Women	Men	Women	Men	Women
Tension–anxiety	.84	.99	.70	*M*	8.57	10.12	7.70	8.43	7.22	8.64
				*SD*	5.06	5.59	4.55	5.92	4.73	5.46
Depression–dejection	.87	1.64	2.57	*M*	6.84	9.86	6.32	9.06	6.22	8.26
				*SD*	7.20	10.34	8.14	10.77	7.67	9.13
Anger–hostility	.79	1.45	2.39	*M*	10.59	10.24	9.14	9.72	8.20	9.99
				*SD*	9.51	9.40	8.16	9.93	7.94	8.68
Vigor–activity	.78	–.26	–.44	*M*	19.96	15.36	21.20	17.32	19.08	14.75
				*SD*	6.39	7.22	6.63	6.35	6.29	6.18
Fatigue–inertia	.85	.77	.28	*M*	8.35	8.66	6.21	8.16	6.09	8.36
				*SD*	5.40	5.04	4.15	4.86	4.61	5.30
Confusion–bewilderment	.80	.77	.24	*M*	9.25	10.40	7.18	8.38	7.08	8.36
				*SD*	4.81	4.71	4.17	4.61	3.80	4.72
Negative mood state	.89	1.26	1.53	*M*	43.61	49.28	36.56	43.75	34.81	43.61
				*SD*	27.44	31.28	25.55	32.04	24.70	29.52

### Age and gender differences for exercise dependence symptoms

The 2 (gender) × 3 (age group) ANOVA for total EDS (dependent variable) showed a significant main effect of gender, *F*(1, 403) = 15.34, *p* < .001, η_p_^2^ = .03 and age, *F*(2, 403) = 6.02, *p* = .003, η_p_^2^ = .029 (see Table 2). The interaction effect between age group and gender was not significant, *F*(2, 403) = .17, *p* = .84, η_p_^2^ = .001. Examination of the mean scores revealed that the men had higher EDS scores than the women. *Post-hoc* analyses for age revealed that the middle-aged adults had lower exercise dependence scores than the adult group (25–44 years), Tukey-HSD, *p* = .02 and then the young adult group (18–24 years), Tukey-HSD, *p* = .005.

In the light of the significant effects on the total EDS score, a MANOVA was conducted with the seven EDS subscales as dependent variables. Results showed that there was a significant main effect of age group [Wilks' Lambda = .91, *F*(14, 794) = 2.62, *p* = .001, η_p_^2^ = .04] and gender [Wilks' Lambda = .91, *F*(7, 397) = 5.47, *p* < .001, η_p_^2^ = .08]. The interaction, however, between age and gender did not reach significance [Wilks' Lambda = .96, *F*(14, 794) = 1.06, *p* = .391, η_p_^2^ = .018].

Univariate analyses revealed significant main effects of age group [Wilks' Lambda = .91, *F*(14, 794) = 2.62, *p* = .001, η_p_^2^ = .044], for *tolerance, F*(2, 403) = 8.11, *p* < .001, η_p_^2^ = .04, *reduction in other activities, F*(14, 794) = 4.59, *p* = .01, η_p_^2^ = .02, *time, F*(14, 794) = 8.91, *p* < .001, η_p_^2^ = .04 and *intention, F*(14, 794) = 4.27, *p* = .01, η_p_^2^ = .02. As well, univariate analyses revealed that the men scored higher than the women on *tolerance, F*(1, 403) = 25.68, *p* < .001, η_p_^2^ = .060, *lack of control, F*(1, 403) = 6.35, p = .01, η_p_^2^ = .016, *reduction in other activities, F*(1, 403) = 5.31, *p* = .02, η_p_^2^ = .01, *time, F*(1, 403) = 14.35, *p* < .001, η_p_^2^ = .03, and *intention, F*(1, 403) = 8.16, *p* = .005, η_p_^2^ = .02.

**Table 4.** Correlation analyses

		1	2	3	4	5	6	7	8	9
1.	Age									
2.	Exercise frequency	–.11*								
3.	Tension–anxiety	–.10*	.01							
4.	Depression–dejection	–.05	–.07	.73**						
5.	Anger–hostility	–.06	.03	.76**	.78**					
6.	Vigor–activity	–.02	.20**	–.29**	–.44**	–.38**				
7.	Fatigue–inertia	–.10	–.02	.69**	.71**	.69**	–.39**			
8.	Confusion–bewilderment	–.17**	–.08	.66**	.65**	.58**	–.23**	.67**		
9.	Negative mood state	–.09	–.03	.87**	.92**	.91**	–.41**	.84**	.78**	
10.	Exercise dependence	–.16**	.34**	.21**	.15**	.25**	.10*	.20**	.16**	.22**

*Note:* * *p* < .05; ** *p* < .01.

*Post-hoc* analyses for age indicated no significant differences between the young adult group (18–24 years) and the adult group (25–44 years) for the EDS subscales. The young adult and adult age groups reported higher levels of *tolerance* (young adult: Tukey-HSD, *p* = .001; adult: Tukey-HSD, *p* = .005), and *time* (young adult: Tukey-HSD, *p* =. 005; adult: Tukey-HSD, *p* < .001) compared to the middle-aged adults. Finally, middle-aged adults (45–64 years) reported lower score in *reduction in other activities* (Tukey-HSD, *p* = .01) than the young adult group and lower score in *intention* than (Tukey-HSD, *p* = .01) the adult group.

### Relationship among age, gender, exercise frequency, mood states, and exercise dependence

Pearson correlations were computed among age, gender, exercise frequency, mood states, and exercise dependence symptoms (see Table 4). Small negative correlations were observed between exercise dependence and age. Small to moderate positive correlations were found among exercise dependence with, mood states and exercise frequency. Intercorrelations among negative mood state, tension–anxiety, depression–dejection, anger–hostility, fatigue–inertia, confusion–bewilderment were large (range *r* = .58 to .78) and are presented in Table 3. Small to moderate intercorrelations among negative mood state and vigor–activity (range *r* = .20 to .44) were observed. Furthermore, to explore whether the data were marked by multicollinearity, both variance inflation and tolerance values were examined. Some critical values were found for the negative mood subscale (tension–anxiety, depression–dejection, anger–hostility, fatigue–inertia, confusion–bewilderment) for both variance inflation (from 2.321 to 3.559) and tolerance (from .281 to .431). For this reason and because the negative mood sub-scales (tension–anxiety, depression–dejection, anger–hostility, fatigue–inertia, confusion–bewilderment) measure the same underlying concept, these variables were combined into a single index total scores for the negative mood states that were used when predicting exercise dependence.

Another collinearity analyses for the regression model with the total index score for the negative mood state indicated that the assumption of multicollinearity was not violated. Multiple regression analysis was used to examine whether age (three previous groups of age), gender (1 = male, 2 = female), exercise frequency, total index of negative mood state and vigor–activity, predicted exercise dependence symptoms (Table 5). The model explained 21.2% of the variance in the Exercise Dependence Scale scores, *F*(5, 403) = 22.89, *p* < .001, *Adj.R*^2^ = .212, with age, *β* = –.09, *p* = .030, gender, β = –.15, *p* = .001, exercise frequency, β = .29, *p* < .001, negative mood, β = .28, *p* < .001, and vigor–activity, β = .12, *p* = .023, being a significant predictor of exercise dependence symptoms. Specifically young age, male, and high levels of exercise frequency, negative mood state, and vigor–activity, significantly predicted higher EDS scores.

**Table 5.** Regression analyses on exercise dependence

Exercise dependence *F*(5, 403) = 22.89, *p* < .001, *Adj.R*^2^= .212		
	β	*t*
Age	–.10	–2.18*
Gender	–.15	–3.32**
Exercise frequency	.29	6.40***
Vigor–activity	.12	2.29*
Negative mood state	.29	5.94***

*Note:* * *p* < .05; ** *p* < .01; *** *p* < .001.

## DISCUSSION

The aim of our study was to examine the prevalence of exercise dependence and the role of age and gender on mood states, exercise frequency, and exercise dependence symptoms. Consistent with our hypothesis we found that age, mood, exercise frequency, and gender predicted exercise dependence. More specifically, we found that the two youngest groups (i.e., young adults and adults) reported higher exercise dependence scores than the oldest group (middle-aged adults), and the women reported lower exercise dependence scores than the men. Implications of our study findings and future research directions are discussed below.

First, our results support previous research, that once controlling for secondary exercise dependence and using a validated exercise dependence measure, that men report more exercise dependence symptoms than women (Berczik et al., 2012). This may be due to the fact that exercise for men is essential for obtaining a muscular physique, whereas women may find that exercise, without simultaneous calorie reduction, may not yield their desired physique (Hausenblas & Fallon, 2002). From a body image perspective, to meet a perceived high societal standard men could develop a “drive for muscularity” while women could develop a “drive for thinness” (McCreary & Sasse, 2000; Vocks, Hechler, Rohrig & Legenbauer, 2009).

Second, we found that exercise dependence symptoms declined with age with the middle-aged adults (aged 45–64 years) reporting lower exercise dependence scores than the adult group (25–44 years) and the young adult group (18–24 years; Edmunds et al., 2006; Sussman et al., 2011; Szabo, 2000). This may be due to the fact that physical activity levels decrease gradually with age (Brunet & Sabiston, 2011; Haskell et al., 2007) and that older adults may be able to regulate their emotions better than younger adults (Birditt et al., 2005; Magai et al., 2006), thus reducing the risk of exercise dependence. Our results, however, are in contrast to Hale et al. (2010) who failed to find age difference in exercise dependence between young adults (18–24 years) and adults (25–55 years). The discrepant age findings can be explained by the wide range that was used by Hale et al. (2010).

Third, we expanded previous research and found that age differences also exist in the EDS subscale. That is, the young adult group and the adult group reported higher levels of *tolerance* and *time* compared to middle-aged adults; and also that middle-aged adults reported lower scores in *reduction in other activities* than young adult group and lower scores in *intention* than the adult group. These results underline how younger adults could be more at-risk for specific symptoms of exercise dependence, and it is likely related to the negative relationship between exercise behavior and age (Brunet & Sabiston, 2011; Haskell et al., 2007; Sallis, 2000).

Fourth, that mood states are related to exercise dependence with negative mood states and vigor–activity predicting exercise dependence symptoms. Several studies have suggested that mood states could play a role in the development or in the maintenance of exercise dependence (Berczik et al., 2012; Hamer & Karageorghis, 2007). Consistent with affect regulation hypothesis for dependence (Hamer & Karageorghis, 2007), physical activity results in improvements in positive mood states and decreased in negative mood states. As the exercise cycle continues, increased amounts of exercise are needed to experience improvement in affect and general mood. Our findings confirm the relationship between mood states and exercise dependence, and also clarify the role of gender and age in the development and maintenance of exercise dependence symptoms.

Finally, we found that the prevalence of individuals at-risk (4.4%) for exercise dependence is comparable to research conducted in several countries (Costa et al., 2012; Lindwall & Palmeira, 2011; Symons Downs et al., 2004), and confirms that using a psychometrically well-validated instrument and after controlling for secondary exercise dependence the prevalence of exercise addiction ranges between 3% and 5% of the exercising population (Berczik et al., 2012; Mónok et al., 2012; Sussman et al., 2011). Furthermore, confirming previous research most of the at-risk participants were men and in the adult age group (25–44 years). Our results reveal that adulthood is the critical age for the development of exercise dependence. The first symptoms of exercise dependence may develop before adulthood, but the risk manifestation of exercise dependence seems to happen during young adulthood. Longitudinal studies tracking exercise behavior and exercise dependence symptoms from childhood into adulthood are needed to verify this statement.

Another aspect to take into consideration is that EDS-R is a screening device to distinguish among at-risk, nondependent symptomatic, and nondependent asymptomatic individuals, and is not a diagnostic instrument to define a diagnosis of exercise dependence. Exercisers classified as at-risk must undergo clinical interviews and/or medical exams before a formal diagnosis of exercise dependence can be established. Furthermore, this study was restricted to the investigation of linear change only, and did not examine nonlinear association or interactions between variables. Future research should also investigate exercise dependence in several different age groups including late adolescents (15–18 years) and senior adults (> 65 years).

Although our study results advance the extant literature, design limitations exist. First, self-reported data for physical activity frequency may have intrinsic limitations such as social desirability (Shephard, 2003). Second, although this is one of the few studies to examine age-related changes on the exercise dependence, results of this study are based on cross-sectional comparisons of participants in different age groups, rather than observations of change as individuals grow older. Use of longitudinal data tends to provide better interpretation, allowing attributions regarding cause or direction of effects. Finally, research is needed on the mediating mechanisms underlying exercise behavior change across the lifespan.

In summary, our study makes several significant contributions to exercise dependence literature by providing the prevalence of exercise dependence in different periods of the adult lifespan and revealing information on at-risk groups for exercise dependence. Furthermore, it is an important first step in understanding differences in exercise dependence symptoms and in the associations between exercise dependence and physical activity behavior and mood states across the adult lifespan. Finally, our study may aid in identifying individuals who may be at-risk for exercise dependence symptoms and, thus help to target and guide the implementation of prevention program across the lifespan.
